# The construction of the multifunctional targeting ursolic acids liposomes and its apoptosis effects to C6 glioma stem cells

**DOI:** 10.18632/oncotarget.19784

**Published:** 2017-08-02

**Authors:** Xue Ying, Yahua Wang, Haolun Xu, Xia Li, Helu Yan, Hui Tang, Chen Wen, Yingchun Li

**Affiliations:** ^1^ School of Pharmaceutical Sciences, Shihezi University, Shihezi 832002, People's Republic of China; ^2^ School of Science, Harbin Institute of Technology (Shenzhen), Shenzhen 518055, People's Republic of China

**Keywords:** glioblastoma, multifunctional targeting ursolic acids liposomes, glioma stem cell, apoptosis, brain glioma-bearing mice

## Abstract

Brain gliomas, one of the most fatal tumors to human, severely threat the health and life of human. They are capable of extremely strong invasion ability. And invasive glioma cells could rapidly penetrate into normal brain tissues and break them. We prepared a kind of functional liposomes, which could be transported acrossing the blood-brain barrier (BBB) and afterwards induce the apoptosis of glioma stem cells. In this research, we chose ursolic acids (UA) as an anti-cancer drug to inhibit the growth of C6 glioma cells, while epigallocatechin 3-gallate(EGCG) as the agent that could induce the apoptosis of C6 glioma stem cells. With the targeting ability of MAN, the liposomes could be delivered through the BBB and finally were concentrated on the brain gliomas. Cell experiments *in vitro* demonstrated that the functional liposomes were able to significantly enhance the anti-cancer effects of the drugs due to promoting the apoptosis and endocytosis effects of C6 glioma cells and C6 glioma stem cells at the same time. Furthermore, the evaluations through animal models showed that the drugs could obviously prolong the survival period of brain glioma-bearing mice and inhibit the tumor growth. Consequently, multifunctional targeting ursolic acids liposomes could potentially improve the therapeutic effects on C6 glioma cells and C6 glioma stem cells.

## INTRODUCTION

Glioblastoma is one of the most common and severe forms of malignant gliomas. The patients could hardly recover even after the operation, radiotherapy and chemotherapy. Their median survival time is only about 14.6 months. The difficulty which exists in treatments of glioblastoma is a part of small cell subsets (1%) couldn't be killed after being treated with radiotherapy and chemotherapy [1, 2]. And most of gliomas are made up by these cell clusters which are capable of different proliferative potentials and transplanted tumor activities. Some cell subsets called cancer stem cells (CSCs) are capable of self-renewing and differentiation, which are partly existed in heterogeneous cell populations. And they are assumed to be the main reason of tumor recurrence [3, 4].

Accordingly, to develop a drug delivery system that could induce the apoptosis of glioma stem cells is worth studying. Liposomes, hollow spheres that made up by phospholipid bilayer, are able to meanwhile load both hydrophilic and hydrophobic drugs. They could decrease the toxicity and increase the bioavailability of drugs [5, 6]. In present research, we built functional liposomes that could pass through the blood-brain barrier (BBB). Ursolic acids (UA) and epigallocatechin 3-gallate (EGCG) were loaded into liposomes as anti-cancer drugs that could induce the apoptosis of glioma cells and glioma stem cells at the same time. P-amino-phenyl-α-D-manno-pyranoside (MAN) shows good effects on assisting drug's carrier to be penetrated across the BBB [7]. It is reported that MAN has a specific affinity to glucose transporters 1 (GLUT1), which is highly expressed on BBB and brain gliomas [8]. Depending on this advantage, the amount of drugs in the brain could be increased after being penetrated across the BBB. So we modified MAN onto the surface of the liposomes as a targeting ligand in order to play a key role in transporting across the BBB as well as enhance the targeting ability of drug-loaded liposomes.

Ursolic acids (UA) is a kind of pentacyclic triterpenoid that exists in natural plants, which mainly exhibits anti-inflammatory effect and anti-cancer effect [9, 10]. It was reported that [11] the inhibitory effect of ursolic acid to C6 glioma cells was significantly related to its concentration. Meanwhile, the caspase-3, 8 and 9 in BGC-803 cells could be activated by ursolic acid while Bcl-2 were oppositely down-regulated [12]. Based on the way of decreasing tumor proliferation and inducing tumor apoptosis, we could somehow infer that ursolic acid was able to effect on C6 glioma cells and glioma stem cells both *in vitro* and *in vivo*. Through these researches, ursolic acid could be developed to potentially treat with C6 glioma cells and glioma stem cells. Meanwhile, the low bioavailability and poor water solubility limit its application in many ways of pharmaceutical field and influence the anti-cancer effect.

Epigallocatechin-3-gallate (EGCG), a hydrophilic component that is extracted from Chinese green tea, highly exists in catechin. EGCG has anti-angiogenic activity and anti-cancer effect [13]. The literatures reported that EGCG could increase the expression of pro-apoptotic protein Bax in human glioma cells, decrease the expression of Bcl-2 protein (Inhibitor of apoptosis protein) and enhance the cleavage of substrates at downstream PARP (DNA repair enzyme, a main member of Caspase) [14, 15]. In addition, EGCG also exibits the inhibitory effects on human glioma stem cells, and partly induced by cell apoptosis [16].

**Figure 1:**
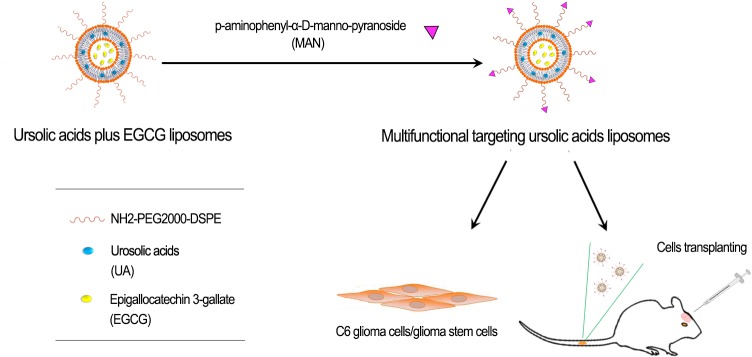
Experimental methods description Prepare multifunctional targeting ursolic acids liposomes, and further test its antitumor effects against C6 glioma cells and glioma stem cells *in vitro* and *in vivo*.

It was supposed to build a kind of multifunctional targeting liposomes loaded with ursolic acids plus EGCG in this research. Later on, several effects would be tested, which were separately based on the apoptosis effects to C6 glioma cells and glioma stem cells *in vitro* and therapeutic effects *in vivo*, as described in Figure 1.

## RESULTS

### Characterization of targeting liposomes

**Figure 2:**
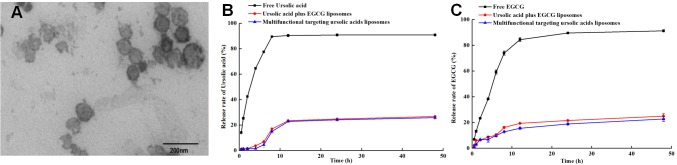
Characterization of multifunctional targeting ursolic acids liposomes **(A)** Transmission electron microscope (TEM) picture of multifunctional targeting ursolic acids plus EGCG liposomes. **(B** and **C)** represent the release rates of ursolic acids and EGCG from three different formulations in PBS solution containing 2% sodium dodecyl sulfate. Data are presented as the mean ± standard deviation, and each assay was repeated in triplicate.

The encapsulation efficiency of ursolic acids and EGCG loaded by multifunctional targeting ursolic acids liposomes were 78.90±0.57% and 75.53±0.56% through estimating by HPLC, respectively. And the connection efficiency of MAN was 32.95±1.06%. Furthermore, the particle size of targeting liposomes was 109.03±0.34 nm, and their zeta potential was −12.30±0.10 mV (Table 1). The electrostatic repulsion that properly exists among the liposomes could make them more stable and hard to aggeragate. According to the results of Figure 2A, the morphology of liposomes were spherical, roundness and smoothly surfaced, and size distribution of liposomes was uniform.

**Table 1:** The paticle size and Zeta potential of different liposomes (n=3)

Groups	Particle sizes (nm)	PDI	Zeta potential (mV)
Blank liposomes	113.83±0.12	0.209±0.07	−8.88±0.45
Ursolic acid plus EGCG liposomes	115.03±0.58	0.203±0.01	−8.49±0.54
Multifunctional targeting ursolic acids liposomes	109.03±0.34	0.181±0.14	−12.30±0.10

Figure 2B showed that the release efficiency of free ursolic acids was 90.94±1.10% at 48h, which was faster than multifunctional targeting ursolic acids liposomes (25.88±1.21%). As it could be seen in Figure 2C, the rank of release efficiency of EGCG was free EGCG(91.25±0.78%)>ursolic acids liposomes plus EGCG liposomes(24.98±1.81)> multifunctional targeting ursolic acids liposomes(22.75±1.7%).

### Identification of C6 glioma stem cells

**Figure 3:**
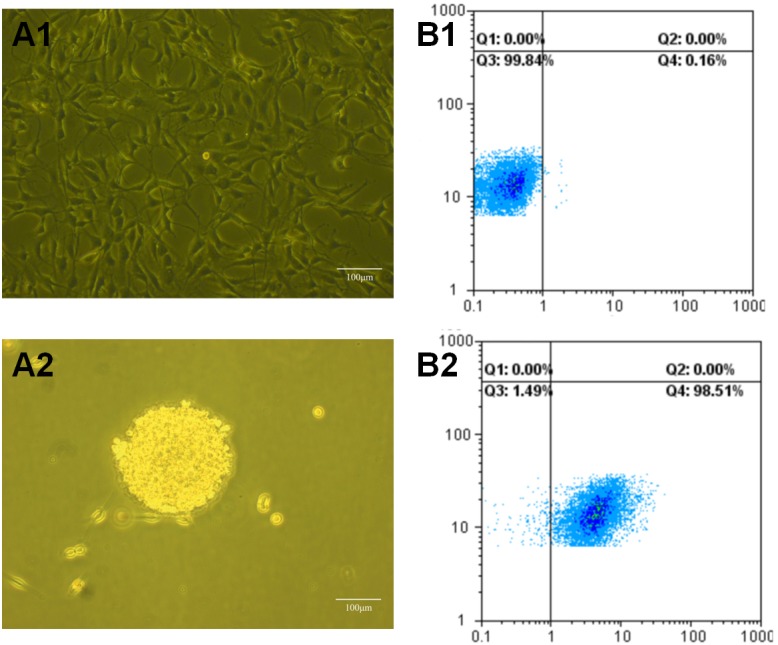
Images of C6 glioma cells and C6 glioma stem cells under microscope and FAScan flow cytometry **(A1)** Images of C6 glioma cells under microscope; **(A2)** images of glioma stem cells(GSCs) under microscope; **(B1)** glioma stem cells spheroids treated as isotype controls under FAScan flow cytometry; **(B2)** glioma stem cells spheroids stained with anti-mouse/rat nestin phycoerythrin antibiodies under FAScan flow cytometry.

Figure 3 separately showed C6 glioma cells (Figure 3A1) and C6 glioma stem cells that grown in serum-free medium (Figure 3A2). Figure 3B1-3B2 demonstrated that the expression rate of Nestin in C6 glioma stem cells was 98.51% compared to isotype contrast. The results displayed that serum-free medium was better for highly expression of Nestin in C6 glioma stem cell spheres, which proved this culture condition to be a proper way for the growing and proliferation of C6 glioma stem cells.

### Antiproliferative effect on C6 glioma cells and C6 glioma stem cells (SRB)

**Figure 4:**
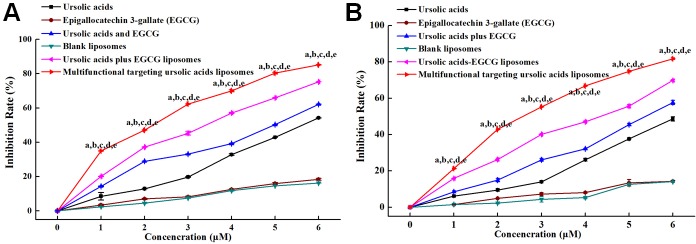
The cytotoxic effects to cells after being treated with various formulations **(A)** The cytotoxic effects to C6 glioma cells; **(B)** the cytotoxic effects to C6 glioma stem cells. 0, 1, 2, 3, 4, 5 and 6 represent that ursolic acids concentration gradient is 0, 0.1, 0.5, 1, 5, 10 and 20 μM, the concentration of EGCG were 0, 0.0164, 0.0819, 0.164, 0.819, 1.637 and 3.275μM.a, P < 0.05, versus blank liposome; b, P < 0.05, versus free EGCG; c, P < 0.05, versus free ursolic acids; d, P < 0.05, versus free ursolic acids and EGCG; e, P < 0.05, versus ursolic acids plus EGCG liposomes.

Figure 4A illustrated that the inhibition effect of free ursolic acids to C6 glioma cells was weak, but it was obviously increased when being combined with EGCG. Multifunctional targeting ursolic acids liposomes showed the most significant antiproliferative effect compared to that of others controls. Figure 4B demonstrated that the antiproliferative effect to C6 glioma stem cells was stronger after combining ursolic acids with EGCG in comparison with others controls. The rank of antiproliferative efffects was multifunctional targeting ursolic acids liposomes> ursolic acids plus EGCG liposomes > free ursolic acids and EGCG > free ursolic acids > free EGCG. According to the results, the combinations of 0.1 μM ursolic acids with 0.0164 μM EGCG, 0.5 μM ursolic acids with 0.0819 μM EGCG, 1 μM ursolic acids with 0.164 μM EGCG and 5 μM ursolic acids with 0.819 μM EGCG all exhibited synergic effects of antiproliferation against C6 glioma cells. Additionally, the combinations of 10 μM ursolic acids with 1.637 μM EGCG and 20 μM ursolic acids with 3.275 μM EGCG showed additive effects of antiproliferation against C6 glioma cells. Meanwhile, the combination of 1 μM ursolic acids and 0.164 μM EGCG illustrated synergic effect of antiproliferation against C6 GSCs. Furthermore, the combinations of 0.1 μM ursolic acids with 0.0164 μM EGCG, 0.5μM ursolic acids with 0.0819 μM EGCG, 5 μM ursolic acids with 0.819 μM EGCG, 10μM ursolic acids with 1.637μM EGCG and 20μM ursolic acids with 3.275 μM EGCG all exhibited additive effects of antiproliferation against C6 glioma stem cells. All the results demonstrated that the combination of ursolic acids and EGCG could inhibit the proliferation of C6 glioma cells and C6 glioma stem cells in synergic and additive ways.

### Effect of liposome on apoptosis of C6 glioma cells and C6 glioma stem cells

**Figure 5:**
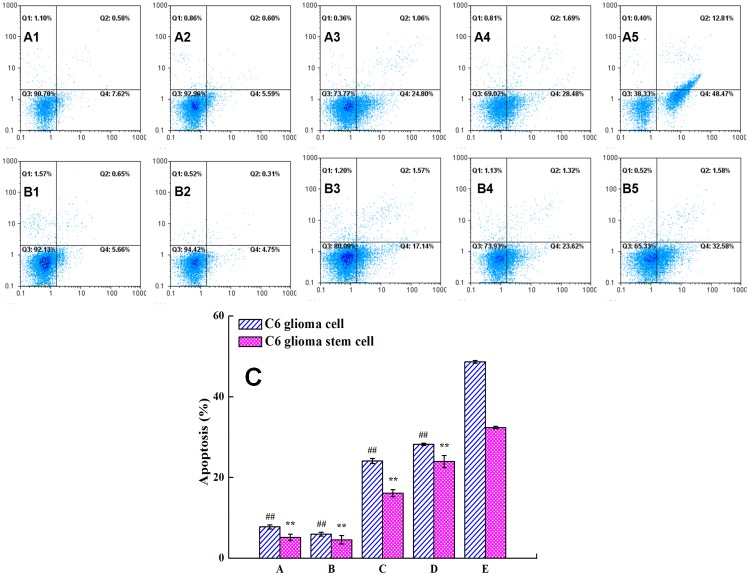
Apoptotic effects against C6 glioma cells and GSCs were obtained by FAScan flow cytometer following varying fomulations Images **(A1-A5)** represent the apoptotic population were treated varying fomulations in C6 glioma cells. Images **(B1-B5)** represent the apoptotic population were treated varying fomulations in C6 glioma stem cells. **(A1, B1)** free EGCG; **(A2, B2)** free ursolic acids; **(A3, B3)** free ursolic acids plus EGCG; **(A4, B4)** ursolic acids plus EGCG liposomes; **(A5, B5)** multifunctional targeting ursolic acids liposomes. Figure 5C was obtained by calculating the percentage of apoptotic population from three independent experiments on C6 glioma cells and glioma stem cells, respectively. Datas are presented as the mean ± standard deviation (SD). ##, P < 0.01, versus multifunctional targeting ursolic acids liposomes in C6 glioma cells; **, P < 0.01, versus multifunctional targeting ursolic acids liposomes in C6 glioma stem cells.

Figure 5A1-5A5 interpreted that the apotosis effect on C6 glioma cells was 48.47% for multifunctional targeting ursolic acids liposomes, 28.48% for ursolic acids plus EGCG liposomes, 24.80% for free ursolic acids and EGCG, 7.62% for free EGCG, and 5.59% for free ursolic acids, separately. And, in Figure 5B1-5B5, the apotosis effect to C6 glioma stem cells was 32.43% for multifunctional targeting ursolic acids liposomes, 23.62% for ursolic acids plus EGCG liposomes, 17.14% for free ursolic acids and EGCG, 5.14% for free EGCG, and 4.75% for free ursolic acids, successively. The results demonstrated that the apoptosis effects of drug-loaded liposomes to C6 glioma cells were stronger than that of C6 glioma stem cells. Thus, the multifunctional targeting ursolic acids liposomes had the most obvious apoptosis effect to both of C6 glioma cells and C6 glioma stem cells.

### Intracelluar localization and apoptosis effects in C6 glioma stem cells detected by laser confocal scanning microscopy

**Figure 6:**
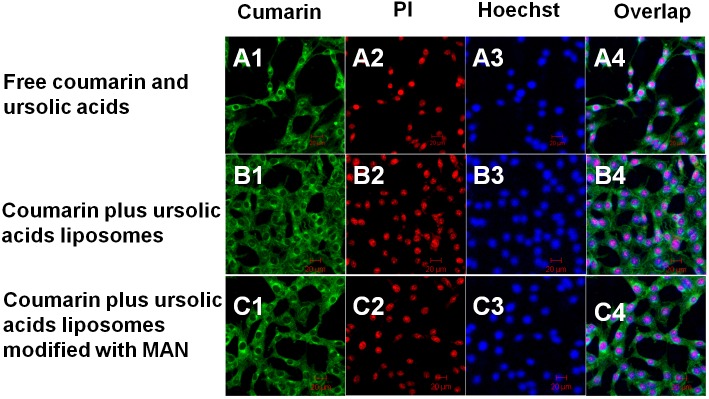
Cellular uptake and distribution in the C6 glioma stem cells Images A–C represents drug distribution in the C6 glioma stem cells by being applied with various formulations and detected by laser scanning confocal microscopy. **(A1–C1)** The green color indicates the drug in the glioma stem cells after applying free coumarin and ursolic acids (A1), coumarin plus ursolic acids liposomes (B1), and coumarin plus ursolic acids liposomes modified with MAN (C1), respectively. **(A2–C2)** The red color shows the drugs induced apoptosis of the C6 glioma stem cells. **(A3–C3)** The blue color refers to the distribution of nucleus after being stained with Hoechst 33258. **(A4–C4)** Overlapping images of (A1–A3), **(B1–B3)** and (C1–C3). Magnification ×20.

Figure 6 illustrated that the liposomes could be penetrated into the cells under applying coumarin as the marker and multifunctional targeting coumarin liposomes could be better penetrated into the cells compared to that of other two groups. From the results we could conclude that it was able to observe the apoptotic morphology of the cells after being stained by PI. And there were some size and morphology differences of the fragments among the nucleus. The nucleus showed more significant split in group of multifunctional targeting coumarin liposomes in comparison with other two groups.

### Evaluation on glioma-bearing mice model

#### Tumor inhibition rates

**Figure 7:**
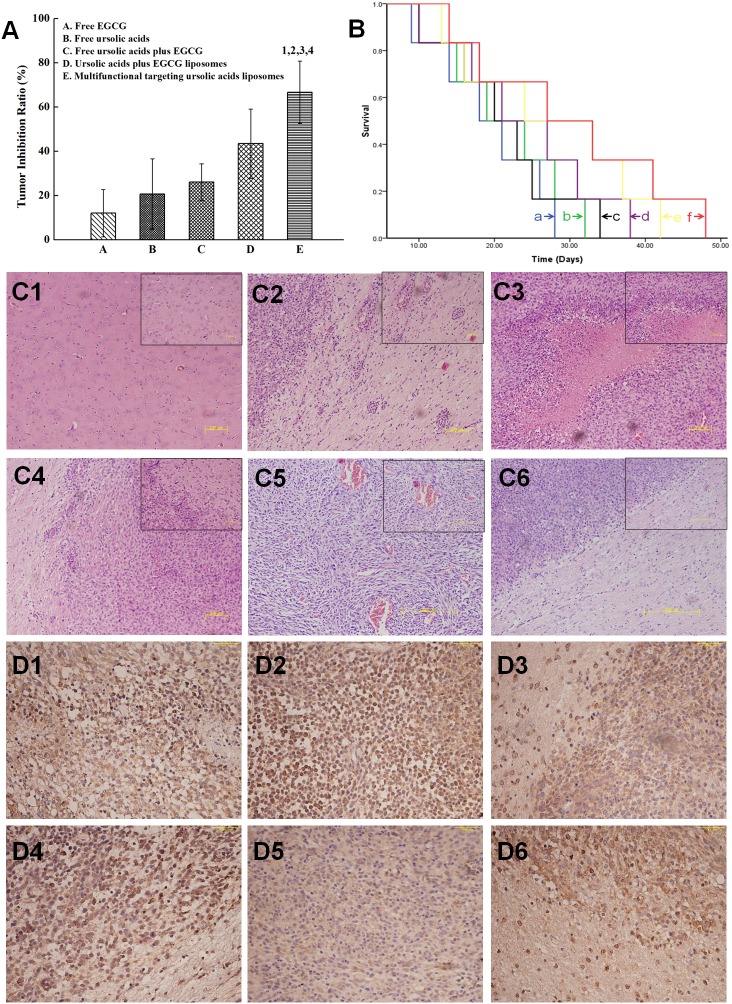
The inhibitory effects in glioma-bearing ICR mice after treatment with varying formulations **(A)** Inhibitory rates of gliomas; 1, P < 0.05, versus free EGCG; 2, P < 0.05, versus free ursolic acids; 3, P < 0.05, versus free ursolic acids plus EGCG 4, P < 0.05, versus ursolic acids plus EGCG liposomes. **(B)** Kaplan-Meier survival curves; **(C)** images of HE staining. **(C1)** The normal brain tissues; **(C2)** the glioma cells and cell subsets were distributed at the margin of glioma tissues; **(C3)** the hemorrhagic and necrotic areas; **(C4)** glioma tissue had no significant margins and capsules with normal tissues; **(C5)** there were abundant microvessels; **(C6)** multifunctional targeting ursolic acids liposomes. **(D)** CD133 immunohistochemical staining. **(D1)** Physiological (0.9%) saline; **(D2)** free ursolic acids; **(D3)** free EGCG; **(D4)** free ursolic acids plus EGCG; **(D5)** ursolic acids plus EGCG liposomes; **(D6)** multifunctional targeting ursolic acids liposomes.

Figure 7A showed that the rank of tumor inhibition rates at day 18 was multifunctional targeting ursolic acids liposomes (66.69±14.1%) > ursolic acids plus EGCG liposomes (43.45±6.67%) > free ursolic acids plus EGCG (23.06±8.28%) > free ursolic acids (20.69±6.87%) > free EGCG (12.07±10.69%). Therefore, the inhibition rate of multifunctional targeting ursolic acids liposomes had the most significant difference compared to others.

#### Survival curves

According to the Kaplan-Meier survival curves of brain glioma-bearing mice arising from glioma stem cells (Figure 7B), the results of median survival curves were multifunctional targeting ursolic acids liposomes(32 days) > ursolic acids plus EGCG liposomes (28 days, *p*=0.046) > free ursolic acids and EGCG (24 days, *p*=0.031) > free ursolic acids (21 days, *p*=0.024) > free EGCG (20 days, *p*=0.020) > physiological saline (18 days, *p*=0.016). From that we know, the survival time of multifunctional targeting ursolic acids liposomes was obviously higher than that of physiological saline.

#### HE staining

Figure 7C1 was the image of normal brain tissues. These cells were well-distributed, without shrinkage, and the nucleus could be easily found. Furthermore, there were some of glioma cells and cell subsets, which mostly shaped oval and were able to proliferated around blood vessels quickly (Figure 7C2), were partly distributed at the margin of glioma tissues. Some hemorrhagic and necrotic areas could be discovered at glioma tissues (Figure 7C3). Glioma tissue had no significant margins and capsules with normal tissues (Figure 7C4). The cell subsets that existed around normal brain tissues were apparently decreased. There were abundant microvessels distributed in glioma tissues, where lots of red blood cells were fully existed (Figure 7C5). Meanwhile, in group of multifunctional targeting ursolic acids liposomes (Figure 7C6), it could be discovered that gliomas were invasively growing toward brain tissues and a clear margin could be seen at the boundary of them.

#### CD133 immunohistochemical staining

In group of physiological saline, the cytoplasmic of glioma cells were obviously stained (Figure 7D1). The cytoplasmic of glioma stem cells were more stained in group of free ursolic acids (Figure 7D2). In group of free EGCG (Figure 7D3), some dispersedly distributed glioma stem cells could be found. The margin of gliomas showed infiltrative growth in group of free ursolic acids and EGCG (Figure 7D4). The results showed that the total amount of glioma stem cells in group of ursolic acids plus EGCG liposomes was significantly more than that of other groups, such as the group of free ursolic acids, free EGCG and free ursolic acids plus EGCG (Figure 7D5). The amount of glioma stem cells was estimated based on CD133 staining. Some hollows exhibited in part of glioma tissues, which may be caused by drug-loaded liposomes killing glioma stem cells (Figure 7D6).

## DISCUSSIONS

Chemotherapy is considered as one of the major ways to treat cancer in clinic. Nowadays, the main problems that limit the clinical application of normal anti-cancer drugs were their low concentration at tumor sites and severe side effects [17, 18]. So the targeting delivery of anti-cancer drugs is now a focus in scientific research. Liposomes are able to load not only hydrophobic drugs but hydrophilic drugs. The hydrophobic drug could be loaded into the phospholipid bilayer, and hydrophilic drug could be encapsulated into the vehicle. So that the interaction of these two drugs could be avoided and therefore enhance their physical ability. In this research, two different drugs were loaded into liposomes in order to decrease their side effects and enhance the targeting ability.

The main problem that exists in the processing of chemotherapy of anti-brain tumors is how to be delivered across the BBB and finally induce the apoptosis of tumors. It is reported [19, 20] that modification with MAN contributes to the transport of the liposomes across the BBB and afterwards increase their amount in the brain due to its specific affinity to GLUT1, which highly express on BBB and tumors. In this experiment, the drug delivery system modified with MAN was built basing on the high expression of MAN in brain microvascular endothelial cells and the targeting ability to its receptor on brain gliomas. This system could assist the delivery across the BBB, increase its concentration in tumor tissues, decrease the damage to normal brain tissues and enhance its therapy effects.

The stability of liposomes is one of the major problem in the processing of preparing liposomes. Accordingly, liposomes with the particle size less than 70 nm are tended to be captured by liver cells. On the contrary, liposomes larger than 300 nm are mainly accumulated at spleen. And the liposomes are more likely to be remained in blood circulation if their particle size is between 70 nm and 200 nm. The particle size of the drug released system built in this experiment is between 100 nm and 200 nm, which means that the drugs loaded in liposomes will be released in a stable manner and the drug blood concentration will be high in order to make better effects in longer duration. The transmembrane leakage of positive charge and proton as well as the aggregation of liposomes could be decreased if the liposomes are negatively charged [21, 22]. Meanwhile, the stability of normal liposomes is significantly enhanced after modifying with PEG2000-DSPE chain. In addition, the Van der Wals forces among the plasma protein and liposomes could also be reduced because of PEG2000-DSPE chain. Therefore, liposomes are able to escape from being captured by monocytes phagocytic system in the body, and their blood circulation time could be prolonged.

In recent years, it is reported [23, 24] that the primary reason of the failure and recurrence of tumor treatments is cancer stem cells. They are capable of drug resistance, self-renewal and potential of proliferation and differentiation. These capabilities play a critical role in all the stages of tumor growing, containing occurrence, development, recurrence and metastasis, even though they are few. So the targeting treatment to cancer stem cells is consequently a way out of this situation. With the further studying on this, a lot more markers of cancer stem cells have been discovered. Thus, CD133 and Nestin are two wildly using markers of brain glioma stem cells [25]. Research elucidated that [26] high expression of Nestin^+^ performed in glioma spheres cultured with growth factor, while CD133^+^ are obviously detected under confocal microscope after immunocytochemical staining. Research proved that [27, 28], as a cell subsets, this kind of cells are capable of sphere-forming ability, rapid proliferation and differentiative ability. In this study, the monoclonal C6 glioma stem cell spheres were incubated by suspended growth method and identified by flow cytometry using Nestin. And the results showed that the Nestin expression level was 98.51%, which demonstrated that this preparation method of monoclonal C6 glioma stem cell spheres met the requirement of this research.

Researches had proved that [29] EGCG could induce cell apoptosis by inhibiting the phosphoralation and expression of AK1. It also illustrated that [30, 31] apoptosis effects could be induced while EGCG prohibiting TNF-α induced NF-κB/p65 from being delivered into nucleus. Additionally, EGCG is also capable of inducing cancer stem cells to apoptosis by restraining the expression of Bcl-2, followed by activating Caspase-9 and its downstream Caspase-3 [32]. In this experiment, the apoptosis effect of C6 glioma cells and C6 glioma stem cells induced by the liposomes modified with MAN was estimated by flow cytometry. Results illustrated that these effects were obviously stronger after combining EGCG with ursolic acids. After being detected by flow cytometry and observed by confocal microscope, the result showed that had significantly higher apoptosis effects to C6 glioma cells and C6 glioma stem cells compared to others. The possible reason might be as follows. The NF-κB (p65) was downregulated, which promoted the ratio of Bax/Bcl-2. After that, the Caspase-3 was activated and finally induced tumor cells to apoptosis [33, 34].

From the experiments *in vitro*, multifunctional targeting ursolic acids liposomes had stronger inhibitory effects to C6 glioma cells and C6 glioma stem cells compared to others. The possible reason of the antiproliferative effects against C6 GSCs induced by targeted liposomes might be as follows. Ursolic acids could inhibit the migration and invasion of tumor stem cells through downregulating MMP-2 and upregulating TIMP-2 [35], while EGCG could inhibit STAT3 signaling pathway [36]. After the combination of ursolic acids and EGCG, the antiproliferative effects were significantly increased through the restraining of two signaling pathways. According to the literature, ursolic acids is able to arrest the cell cycle at G2 phase by activating G2/M checkpoint [37]. So the cells cannot enter mitosis phase. And EGCG could arrest the cell cycle at G0/G1 phase in order to inhibit the synthesis of proteins and RNA [38]. When these two drugs were combined, two phases of cell cycle were arrested. Therefore, the antiproliferative effects were stronger compared to free drugs.

The glioma-bearing mice models in this experiment were built by implanting C6 glioma stem cells into their brain tissues. C6 glioma stem cells had stronger invasive potential and ability to promote gliomas growth compared to C6 glioma cells. These gave a reference to future research about the recurrence of gliomas and biological characteristics of their growth process. The tumor-formation rate of glioma-bearing mice models was high and the gliomas grow well. In the processing of building animal models, the wound on the exposed skull should keep wet to protect it from being influenced by dryness. And, the needle should be stayed at implanting point for 1 min. Then the needle should be pulled out slowly in order to avoid losing cells. It could also prolong the contacting time of glioma stem cells and targeting brain tissues, thus contribute to increase tumor-formation rate.

CD133 is a kind of transmembrane glycoproteins that exist on human blood stem cells. Recent study discovered that it could express on leukemia cells, brain gliomas, colon cancer cells, prostate cancer cells, liver cancer cells and pancreatic cancer cells, which illustrates that CD133 positive tumor cells are capable of stronger ability of proliferation, differentiation and self-renewal [39, 40]. In this experiment, CD133 was primary antibody (1:50) and antiserum of goat against rabbit was secondary antibody. The margin of tumor tissues was growing invasively, and there were large areas of necrosis and hemorrhage in glioma tissues after being stained with HE. Also, CD133 were highly expressed in cytoplasm.

Experiments in animals illustrated that the median survival time and inhibition rate on gliomas treated by multifunctional targeting ursolic acids liposomes were higher than that of other groups. The possible reason was that the PEG2000-DSPE chain could help multifunctional targeting ursolic acids liposomes from being captured by endothelial system, as well as assisting their delivery acrossing the BBB, and finally target the glioma cells. Meanwhile, ursolic acids and EGCG, as anti-cancer drugs, could also induce the apoptosis of glioma stem cells to improve the therapeutic effects.

## MATERIALS AND METHODS

### Preparation of liposomes

Ursolic acids (Nanjing Zelang Biological Technology Co. Ltd., Nanjing, China) and EGCG (Chengdu Planting Standard Pure Biological Technology Co. Ltd., Chengdu, China) were loaded into liposomes modified with MAN was prepared as multifunctional targeting delivery system. Meanwhile, ursolic acids plus EGCG liposomes were also prepared as controls. All liposomes were prepared on the basis of blank liposomes.

#### Blank liposomes

Egg phosphatidylcholine (EPC, Germany Lipoid company, ShangHai local agent, China), cholesterol (CHOL, Beijing Shuangxuan Microbe Culture Medium Products Factory, Beijing, China), polyethylene glycol 2000 distearoylphosphosphatidylethanolamine (PEG_2000_-DSPE, NOF Corporation, Japan), NH_2_-PEG_2000_-DSPE (NH_2_-PEG_2000_-DSPE, Avanti Polar Lipids, Alabaster, USA) were mixed (68.5:33.6:3.6:1, molar ratio) and gradually filmed under vacuum with a rotary evaporator (EYELA-1000S, EYELA Rikakikai Corporation, Tokyo, Japan). After being hydrated by 250mM ammonium under ultrasonication, the solution was treated with ultrasonic cell disintegrator for 5 min in order to diminish the particle size of the liposomes. The suspensions were separately extruded by polycarbonate membrance (Millipore, Bedford, MA, USA) with a pore size of 400 nm and 200 nm for every three times to obtain the blank liposomes.

#### Ursolic acids plus EGCG liposomes

The ursolic acids and EGCG were dissolved in methanol with a drug-lipid ratio about 1:20 and 1:15, respectively. The followed procedures were the same with blank liposomes. Finally, the ursolic acids plus EGCG liposomes were obtained.

#### Multifunctional targeting ursolic acids liposomes

Multifunctional targeting ursolic acids liposomes were prepared as targeting liposomes on the basis of ursolic acids plus EGCG liposomes. In brief, with the help of glutaraldehyde as the coupling agent, the MAN (Sigma–Aldrich Corporation, Beijing local agent, China) was binded to the amino of NH_2_-PEG_2000_-DSPE chain, which was modified on the surface of the ursolic acids plus EGCG liposomes. The specific procedures were: mixed the liposomes with MAN, followed by slowly adding glutaraldehyde to a final concentration about 15 mM and incubated under room temperature for 5 min. Uncoupled MAN and glutaraldehyde were removed by dialysis against PBS (pH 7.4) and finally obtained multifunctional targeting ursolic acids liposomes.

To evaluate the coupling efficiency of MAN, the connecting efficiency of MAN was detected by trinitro-benzene-sulfonic (TNBS, Biodee Biotechnology Co., Ltd., Beijing, China) method after treated liposome suspensions with TNBS as chromogenic agent and tested the NH_2_ that hadn't been binded to the chain NH_2_-PEG2000-DSPE on the multifunctional targeting ursolic acids liposomes [41]. The absorbance of the final solution was estimated by a UV spectrophotometer (UV-2401PC, Shimadzu Technologies Inc., Cotati, CA, Japan) at 320 nm contained with a blank group of distilled water. The coupling efficiency of MAN on the liposomes was estimated with the formula CE_MAN_ = (1−A_uncoupled_ /A_total_) × 100%, where CE_MAN_ is the coupling efficiency of MAN, A_uncoupled_ is the absorbance of uncoupled amino groups of NH_2_–PEG_2000_–DSPE on the liposomes after modifying with MAN, and A_total_ represented the absorption of NH_2_ on the chain NH_2_-PEG_2000_-DSPE before modified liposomes with MAN.

### Characterization of the liposomes

#### Encapsulation efficiency (EE)

The concentration of ursolic acids and EGCG in the liposomes were determined by a high-performance liquid chromatography (HPLC Agilent Technologies Inc., Cotati, CA, USA) system with an ODS–SP column (250 mm × 4.6 mm, 5 μm). The ursolic acids-loaded liposomes and free drugs were separated by Sephadex G-50 column chromatography. The amount of unloaded EGCG was eliminated by dialysis method.

The encapsulation efficiency was calculated by the formula: EE = W_loaded_/W_total_×100%, where EE referred to the encapsulation efficiency of ursolic acids and EGCG, W_loaded_ and W_total_ separately represented the amount of these two drugs that loaded by liposomes and total amount of them in the liposome suspensions before dialysis.

#### Morphology and particle size

The liposomes were diluted by deionized water and stained by 3% phosphotungstic. The morphology was detected by transmission electron microscope (600×) (TEM, JEM-ARM200F, JEOL, Japan). The average particle size and polydispersity index (PDI) were tested by light scattering apparatus (Malvern instruments, Ltd, UK).

#### Drug release *in vitro*

*In vitro* release of ursolic acids formulations were performed by dialysis against the release medium (PBS containing 2% sodium dodecyl sulfate) with a shaker at a rate of 100 rpm at 37°C. The cumulative release percentage of ursolic acids and EGCG were calculated at different time points according to the following formula: R=(W_t_/W_total_)×100%, where R is the drug release rate (%), W_t_ is the measured amount of drug at each time point in the dissolution medium, W_total_ is determined amount of drug prior to dialysis.

### Cell culture and identification of C6 glioma stem cells

#### Culture of C6 glioma cells

C6 glioma cells (Institute of Sciences, Shanghai, China) were grown in Dulbecco's modified Eagle's medium (DMEM, high glucose, Gibco Biotech Co., Ltd., Beijing local agent, China) supplemented with 10% (volume per volume) heat-inactivated fetal bovine serum (Hangzhou Evergreen Company, Hangzhou, China), 100 U/mL of penicillin, 100 μg/mL of streptomycin (Gibco Biotech Co., Ltd., Beijing local agent, China) and maintained in a humidified atmosphere at 37°C with 5% CO_2_.

#### Culture and identification of glioma stem cells (GSCs)

After being dissociated by 0.25% trypsin (Gibco Biotech Co., Ltd., Beijing local agent, China), C6 glioma cells were cultured in serum-free DMEM-F12 (Macgene Gen Techology Co., Ltd., Beijing, China) being supplemented with 10 ng/ml basic fibroblast growth factor (bFGF, Macgene Gen Techology Co., Ltd., Beijing, China), 20 ng/ml epidermal growth factor (EGF, Macgene Gen Techology Co., Ltd., Beijing, China) and 2% B27 (Gibco Biotech Co., Ltd., Beijing local agent, China) [42, 43]. Under these conditions, the C6 glioma stem cells grew as non-adherent spherical clusters of cells named as mammospheres. Half of the media was changed every other day. After 5 days, the mammospheres were collected by centrifugation at 1000 rpm for 5 min and further plated in the new medium. The C6 glioma stem cells mammospheres were cultured in serum-free medium under 5% CO_2_ at 37°C.

#### Identification of C6 glioma stem cells

C6 glioma stem cells were cultured in serum-free medium for 14 days and then separated by trypsin in order to obtain stem cell spheres. Washed them by PBS and settled them by 4% paraformaldehyde. Their membrane were ruptured by 0.1% saponin, followed by cultured with Nestin antibody, and their appropriate isotype controls (R&D Systems, Minneapolis, MN, USA) for 30 minutes away from light. After that, the stem cells were washed by PBS three times and analysed with FACScan flow cytometry (Becton Dickinson FACSCalibur, Mountain View, CA, USA) [44, 45].

### Antiproliferative activity agains C6 glioma cells and C6 glioma stem cells

C6 glioma cells and C6 glioma stem cells were seeded into 96-well culture plates and incubated at 37°C till they were able to grow adhering to the wall. The vlume of 10 μL of blank liposomes, free ursolic acids, free EGCG, free ursolic acids and EGCG, ursolic acids plus EGCG liposomes and multifunctional targeting ursolic acids liposomes were separately added to the cell culture wells with a final ursolic acids concentration gradient about 0, 0.1, 0.5, 1, 5, 10 and 20 μM of each group. Correspondingly, the concentration of EGCG were set as 0, 0.0164, 0.0819, 0.164, 0.819, 1.637 and 3.275μM. After 48h, the absorption degree (540 nm) of every well was evaluated by sulforhodamine B (SRB, Sigma, CA, USA) in order to evaluate inhibition rate. Inhibitory rate was calculated by the formula: Inhibitory rate (%) = 1 – (A_540 nm_ for the treated cells/A_540 nm_ for the control cells) × 100%, where A_540 nm_ is the absorbance value. Evaluated whether there is *in vitro* antiproliferative synergic effects agains C6 glioma cells and GSCs while using ursolic acids and EGCG at the same time. The evaluation was based on the formula: Q=E_u+e_/(E_u_+E_e_-E_u_×E_e_), where E_u+e_ was referred to the inhibitory effect of drug combination, E_u_ was referred to the inhibitory effect of ursolic acids and E_e_ was referred to the inhibitory effect of EGCG, respectively. According to the caculation, when the value of Q was in the range of 0.86∼1.15, 1.16∼2.00, 0.55∼0.85 or lower than 0.55, the results was described as additive effect (+), synergic effect (++), antagonistic effect (−) or obvious antagonistic effect (−-).

### Apoptosis effects to C6 glioma cells and C6 glioma stem cells induced by liposomes

C6 glioma cells and C6 glioma stem cells were seeded into 6-well culture plates at a density of 2 × 10^5^ cells/well and grown in serum-containing medium at 37°C with 5% CO_2_. After that, applied formulations to each well with final concentrations of 10 μM ursolic acids and 1.64 μM EGCG. The groups included free ursolic acids, free EGCG, free ursolic acids and EGCG, ursolic acids plus EGCG liposomes, and multifunctional targeting ursolic acids liposomes. After 24 h incubation, treated them by trypsin and centrifuged (1000 rpm/min) for 5 min. Added 1 ml PBS that precooled at 4°C and centrifuged again. Carefully removed the supernatant, followed by gradually added 250 μL binding buffer, 5 μL Annexin V/FITC and 10μL 20μg/ml propidium iodide (PI). Mixed them under room temperature and reacted 15 min. Then, diluted with 400 μL PBS and estimated by flow cytometry.

### Intracelluar localization and apoptosis effects in C6 glioma stem cells detected by laser confocal scanning microscopy

After loading liposomes with coumarin as the fluorescent marker, utilized laser confocal scanning microscopy (LSM 510, Zeiss, Germany) to detect intracelluar localization and apoptosis effects of formulations in C6 glioma stem cells. In details, C6 glioma stem cells were seeded into 12-well cell culture plates at a density of 1×10^4^ cells/well, and grew in serum-containing medium at 37°C with 5% CO_2_ for 24 h. After that, separately added the formulations of free coumarin and ursolic acids, coumarin plus ursolic acids liposomes, and coumarin plus ursolic acids liposomes modified with MAN with final concentrations about 10 μM ursolic acids and 2 μM coumarin. Cultured them for 24 h, settled by 4% paraformaldehyde after being washed by cold PBS for twice, and stained by Hoechst 33258 for 10 min and PI for 5 min. Then the cells were detected by laser confocal scanning microscopy. (The excitation and absorption wavelength of Hoechst is 352 nm and the absorption wavelength is 505-550 nm; The excitation wavelength of PI is 488 nm, and the absorption wavelength is 630 nm).

### Evaluation on animal model bearing C6 glioma stem cells

#### Construction of the model

Imprinting control region (ICR) mice were purchased from Urumqi of Xinjiang Medical University (male, weighed from 18–20 g). All procedures were performed according to guidelines of the Institutional Authority for Laboratory Animal Care of Shihezi University. ICR mice bearing C6 glioma stem cells were prepared as reported previously [46, 47]. The mice were anesthetized via 20% vinyl carbamate (1 g/kg) and then settled on stereotaxis instrument by symmetrically inserting two ear positioning pin deep into both left and right external auditory canals. Disinfected mice forehead by 75% ethanol and paved sterile sheet. Cut the skin along the mice parietal midline, separated soft tissue and periosteum. Drilled a hole that positioned 0.4 mm anterior from the coronal suture and 2.0 mm lateral from the sagittal suture by dental bit. A total amount of 3 μL C6 glioma stem cells (5×l0^4^/μL) were stereotaxically implanted into the right forehead at a depth of 3.0 mm from the brain surface for 1 μL per minute and stayed for 1 min afterwards. Gradually removed the needle and closed the cranial window by bone wax. Sewed up the incision, disinfected the skin and gave intramuscular injection of penicillin.

#### Tumor inhibition rates

The mice were randomly divided into six groups on the tenth day after C6 glioma cells were successfully inoculated. At day-18, four mice from each group were anesthetized, which the brain tissues were settled by 4% paraformaldehyde after the mice were sacrificed. Cut the coronal incision along the puncture point of brain surface inoculation and measured the size of gliomas by the formula: volume = a^2^×b×π/6 (a was the major diameter of the gliomas, b was the minor diameter of the gliomas). The tumor inhibition rates were calculated using the formula: Rv = 1-(Vexp/Vblank), where Rv was tumor inhibition rates, V_blank_ represented the average volume of the gliomas in blank group and V_exp_ referred to the average volume of the gliomas in formulation groups.

#### Detection of survival curves

At the tenth day after successfully establishing the model, the mice were randomly divided into 6 groups (6 rats per group) and separately administrated with physiological saline, free ursolic acids, free EGCG, free ursolic acids and EGCG, ursolic acids plus EGCG liposomes and multifunctional targeting ursolic acids liposomes by intravenous injection (5 mg/kg) for every other day about 4 times. The survival rate was estimated by Kaplan-Meier method using SPSS 17.0, and obtained the survival curves.

#### HE staining and CD133 Immunohistochemical staining

Settled the brain tissues by 4% paraformaldehyde after the mice were sacrificed. Dehydrated using graded ethanol, vitrified by dimethylbenzene and deposited in wax. The specimens of mice were sliced serially in coronal plane with each thickness about 6 μm. The sections were then processed by H&E staining. Immunohistochemical staining of sections and paraffin slips were performed using CD133 (1:50) as primary antibodies and antiserum of goat against rabbit as secondary antibody. Finnaly, the sections were examined under light microscopy.

### Statistics

Data are presented as the mean±standard deviation (SD). One-way analysis of variance (ANOVA) was used to determine significance among groups, after which post hoc tests with the Bonferroni correction were used for comparison between individual groups. A value of P<0.05 was considered to be significant.

## CONCLUSIONS

In this research, the multifunctional targeting ursolic acids liposomes, as novel kind of drug targeting delivery system, were prepared to treat on glioma cells and glioma stem cells. By conjugating a ligand onto the liposome surface for enhancing permeability of drug across the BBB, and by simultaneously coupling another ligand onto the same liposome surface for targeting brain tumor, and modifing the agent of inducing apoptosis on cancer stem cells into the same liposome for anti-tumor stem cell in brain. The liposomes were evaluated by the experiments *in vitro* and in glioma-bearing mice. Multifunctional targeting ursolic acids liposomes could promote antitumor effects and apoptosis on glioma cells and glioma stem cells, and improve the survivals and inhibition rates *in vivo*. Consequently, multifunctional targeting ursolic acids liposomes provide a potentially therapy strategy on curing refractoriness tumor and relapse of treated brain glioma cells and brain glioma stem cells.
